# Case Report: Negative initial endoscopy and 18F-FDG PET/CT may not exclude occult gastric cancer

**DOI:** 10.3389/fonc.2026.1823046

**Published:** 2026-05-29

**Authors:** Zian Jin, Changhong Dong, Kou Wang, Yanting Jiang, Kaiyuan Hui, Xiaodong Jiang

**Affiliations:** Department of Oncology, The Affiliated Lianyungang Hospital of Xuzhou Medical University, Lianyungang, Jiangsu, China

**Keywords:** adnexal mass, occult gastric cancer, peritoneal dissemination, repeat EGD with biopsy, suspected Krukenberg tumor

## Abstract

Gastric cancer with ovarian metastasis (Krukenberg tumor) can mimic primary ovarian malignancy, creating diagnostic uncertainty. We report a female patient with peritoneal dissemination and bilateral adnexal masses, a presentation initially considered more suggestive of primary ovarian malignancy. However, initial EGD showed no definite gastric mass, and 18F-FDG PET/CT revealed no FDG-avid gastric primary lesion. Peritoneal biopsy revealed metastatic adenocarcinoma with a gastrointestinal-favoring immunophenotype, but repeat EGD was initially deferred due to clinical constraints. Empiric ovarian cancer–directed chemotherapy was started, followed by rapid progression. Repeat EGD later revealed an irregular ulcerative lesion at the gastric angle, and biopsy confirmed gastric adenocarcinoma with focal signet-ring cell components.

## Introduction

Gastric cancer is among the most common gastrointestinal malignancies worldwide and remains a leading cause of cancer incidence and mortality (1). Its carcinogenesis and progression constitute a complex, multistep process driven by the interplay of genetic susceptibility and environmental exposures. Although most cases are sporadic, a subset demonstrates familial aggregation or is associated with established hereditary cancer syndromes (2). The clinical presentation of gastric cancer is heterogeneous; early symptoms are often nonspecific, and many patients are diagnosed at an advanced stage. Peritoneal metastasis, in particular, is a frequent pattern of dissemination in advanced gastric cancer and is associated with a dismal prognosis (3). When gastric cancer involves the ovaries and forms a Krukenberg tumor, patients commonly present with adnexal masses, ascites, and elevated CA125 levels, making the distinction from primary ovarian cancer challenging on clinical and imaging grounds (4). Moreover, metastases from other malignancies such as breast cancer to the stomach, which can mimic early gastric cancer, have also been reported, underscoring the need for vigilance and precise discrimination of the primary site in patients with a prior cancer history or atypical presentations (5).

Here, we report a case of advanced gastric cancer that initially presented with peritoneal carcinomatosis and bilateral adnexal masses, closely mimicking primary ovarian malignancy. Despite an initially negative EGD and the absence of a definite FDG-avid gastric lesion on PET/CT, subsequent clinicopathologic reassessment and repeat EGD with biopsy established the diagnosis of poorly differentiated gastric adenocarcinoma with focal signet-ring cell components. This case highlights the need to maintain suspicion for a gastrointestinal primary and to pursue repeat tissue sampling when the clinical, imaging, and pathologic findings are discordant.

## Case presentation

The patient was a 44-year-old woman who developed postprandial epigastric discomfort in January 2025. At our hospital, she underwent EGD and colonoscopy. EGD revealed scattered punctate erosions in the antral mucosa, and biopsy showed mild chronic inflammation with glandular hyperplasia. Colonoscopy demonstrated moderate chronic mucosal inflammation (**Figures 1A, B**). No specific treatment was administered. Approximately 3 months later, she began to experience nausea, vomiting, decreased appetite, and symptoms suggestive of bowel obstruction, including absence of flatus and bowel movements. Contrast-enhanced abdominal CT performed at that time showed thickening of the antral gastric wall; bilateral adnexal mass lesions; ascites; multiple enlarged lymph nodes; and nodules in the greater and lesser omentum, raising suspicion for peritoneal dissemination/metastatic spread (**Figure 2**). Right hydronephrosis was also noted. Tumor markers were markedly elevated, with a CA125 level of 152 U/mL and a CA19–9 level of 743 U/mL (**Table 1**).

**Figure 1:**
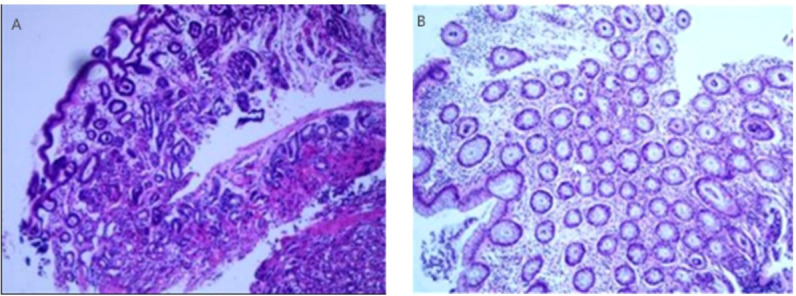
Initial esophagogastroduodenoscopy (EGD) biopsy pathology. **(A)** Gastric antrum biopsy showing mild chronic inflammation with glandular hyperplasia (H&E, ×200). **(B)** Colonoscopic mucosal biopsy showing moderate chronic inflammation (H&E, ×200).

**Figure 2:**
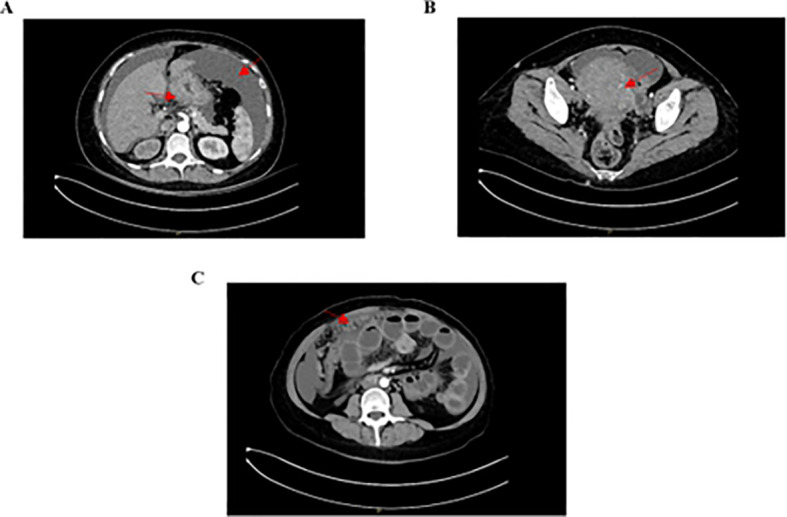
Contrast-enhanced abdominal CT before diagnostic revision. **(A)** Axial contrast-enhanced CT image showing irregular gastric antral wall thickening (arrow) and ascites (arrowhead). **(B)** Axial contrast-enhanced CT image showing an adnexal mass (arrow). **(C)** Axial contrast-enhanced CT image showing nodular thickening of the greater omentum (arrow), consistent with peritoneal metastatic spread.

**Table 1:** Serial changes in serum tumor markers during the disease course.

Time point	CA125 (U/mL)	CA19-9 (U/mL)	CA72-4 (U/mL)	HE4 (pmol/L)
Initial evaluation, April 2025	152	743	12.4	—
Before cycle 1, May 2025	160	1802	13.3	180
Before cycle 2, June 2025	176	3090	14.3	227
After two cycles, June 2025	183	4190	14.6	317
Subsequent reassessment, August 2025	153	3105	13.3	313

She was then admitted to our department. During hospitalization, she developed worsening nausea, vomiting, and obstipation, consistent with bowel obstruction. After consultation with gastrointestinal surgery, exploratory surgery was recommended. During the same hospitalization, she underwent exploratory laparotomy. Intraoperatively, a large volume of ascites was noted, along with diffuse metastatic nodules on the peritoneum and mesentery; biopsies were obtained. A pelvic mass was found to invade the bowel, with marked diffuse small-bowel dilatation. A diverting small-bowel enterostomy was therefore performed to relieve the obstruction. Postoperative histopathology revealed metastatic poorly differentiated adenocarcinoma in the peritoneal nodules. Immunohistochemistry showed CK (partial+), CK7 (3+), CK20 (−), CDX2 (focal+), Ki-67 (40%+), p53 (+, mutant-pattern expression), PAX8 (−), CA125 (−), p16 (3+), ER (−), PR (−), SALL4 (−), CEA (3+), MUC2 (−), MUC5AC (3+), and β-catenin (+). Integrating the immunophenotype with the intraoperative findings, a gastrointestinal primary was considered the leading diagnostic possibility and was prioritized for exclusion.

Further upper and lower endoscopy was planned to clarify a potential gastrointestinal source. However, because she was in the early postoperative period after surgery for bowel obstruction, the patient was concerned that she would not tolerate repeat endoscopy and explicitly declined it. After thorough discussion with the patient and her family, 18F-FDG PET/CT was performed. The scan demonstrated bilateral adnexal soft-tissue thickening, with increased FDG uptake in the right adnexal mass. Mildly increased FDG uptake was also observed in retroperitoneal para-aortic lymphadenopathy, and focal FDG uptake was identified in a pelvic skeletal lesion, suspicious for metastatic involvement. In addition, omental and mesenteric thickening was present but without prominent FDG avidity. No definite abnormal FDG uptake was identified in the gastric antrum on PET/CT or maximum-intensity projection (MIP) images (**Figure 3**).

**Figure 3:**
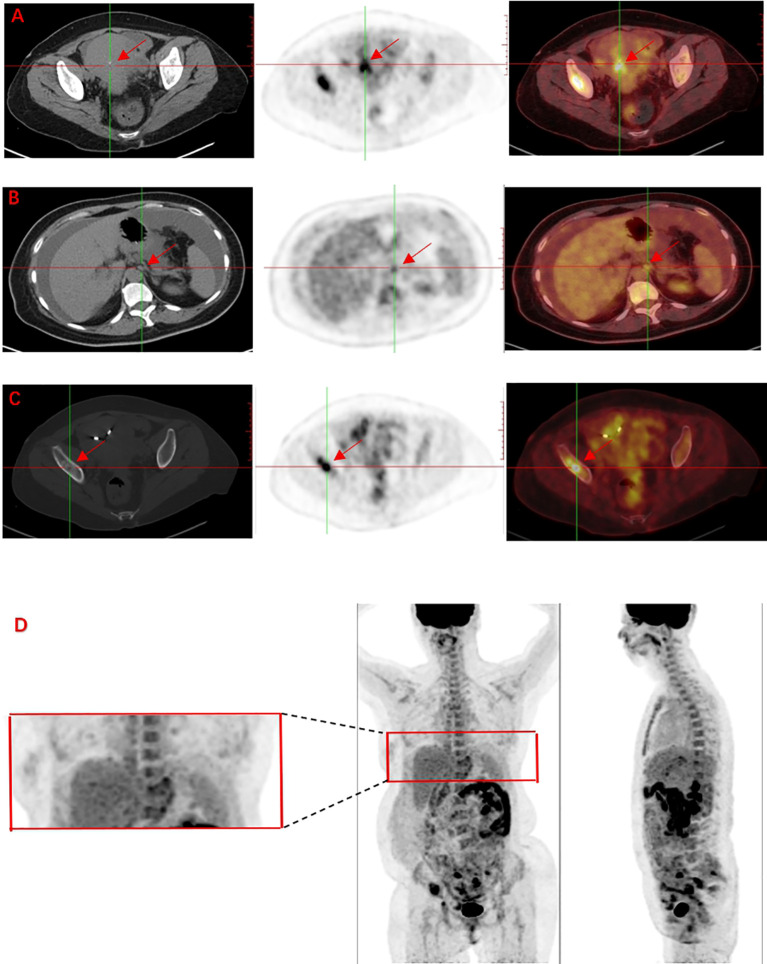
Baseline 18F-FDG PET/CT before diagnostic revision. **(A)** Axial CT, PET, and fused PET/CT images showing focal FDG uptake in the right adnexal mass (arrows). **(B)** Axial CT, PET, and fused PET/CT images showing mildly increased FDG uptake in retroperitoneal para-aortic lymphadenopathy (arrows). **(C)** Axial CT, PET, and fused PET/CT images showing focal FDG uptake in a skeletal metastatic lesion involving the right ilium/right acetabular region (arrows). **(D)** Coronal and sagittal maximum-intensity projection (MIP) PET images showing multifocal abnormal FDG uptake. The enlarged cropped coronal PET image on the left highlights the absence of definite FDG uptake in the gastric antrum.

Based on the dominant bilateral adnexal masses and markedly elevated CA125, the multidisciplinary team considered advanced adnexal malignancy the leading working diagnosis. However, a gastrointestinal primary remained a major competing diagnosis because of the persistent CT evidence of antral wall thickening, the metastatic immunophenotype favoring gastrointestinal origin (e.g., focal CDX2 positivity with PAX8 and CA125 negativity), and the patient’s obstructive presentation. Therefore, the negative 18F-FDG PET/CT findings were not considered sufficient to exclude gastric cancer. Because urgent disease control was required and the patient declined immediate repeat endoscopic evaluation in the early postoperative period, ovarian cancer-directed chemotherapy was initiated only as a temporizing strategy, with a prespecified plan for early diagnostic reassessment, including repeat EGD, if the clinical course proved discordant or gastric wall thickening persisted.

The patient received the first cycle of chemotherapy in May 2025, consisting of nab-paclitaxel 400 mg plus carboplatin 400 mg (cycle 1). In June 2025, she received cycle 2 with the same regimen (nab-paclitaxel 400 mg + carboplatin 400 mg), combined with bevacizumab 800 mg. After two cycles, a follow-up CT suggested disease progression: persistent peritoneal thickening and ascites; worsening right hydronephrosis with suspected ureteral involvement; and suboptimal gastric distension with continued antral wall thickening, with an overall slight increase in tumor burden compared with baseline. Tumor markers also increased, including CA125 183.0 U/mL, CA19-9 4190.0 U/mL, HE4 317.0 pmol/L, and CA72-4 10.60 IU/mL (**Table 1**).

Given radiographic progression after two cycles, continued elevation of tumor markers, and persistent antral wall thickening on CT, repeat endoscopic evaluation was again recommended. The patient agreed and subsequently underwent repeat EGD, which revealed an irregular ulcerative lesion at the gastric angle; biopsy was performed. Histopathology confirmed poorly differentiated adenocarcinoma, with focal signet-ring cell carcinoma components (**Figure 4**).

**Figure 4:**
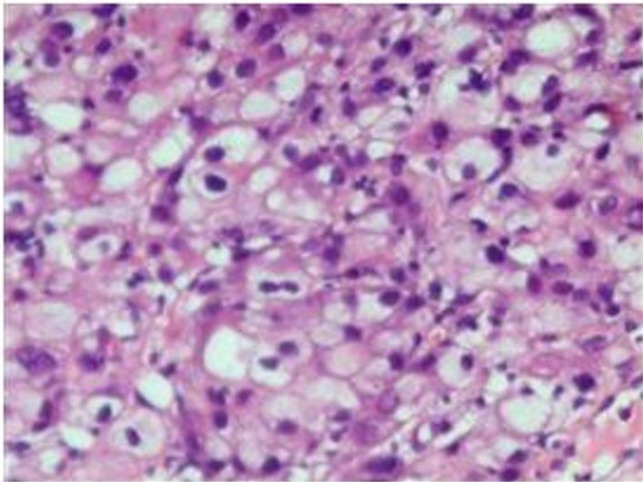
Pathological confirmation of gastric cancer on repeat EGD. Gastric angle biopsy demonstrating poorly differentiated adenocarcinoma with a signet-ring cell component (H&E staining, ×400).

Immunohistochemistry of the gastric biopsy showed CK (3+), CK7 (2+), CDX2 (2+), p53 (mutant-pattern expression), Ki-67 (40% in hot spots), MUC5AC (3+), MUC6 (focal+), PAX8 (−), CA125 (−), Villin (3+), HER2 (1+), and PD-L1 CPS 0. MSI/dMMR testing was not performed in this case. Following multidisciplinary discussion (gastroenterology, radiology, pathology, and oncology), the diagnosis was revised to stage IV gastric cancer with suspected ovarian metastases (suspected Krukenberg tumor).

During this period, the patient developed hydronephrosis with renal insufficiency and underwent percutaneous nephrostomy for urinary drainage. Systemic therapy with immunotherapy combined with chemotherapy was recommended; however, the patient initially declined immunotherapy and requested chemotherapy alone. Considering her renal dysfunction and the potential nephrotoxicity of platinum agents, she was treated with nab-paclitaxel plus fluorouracil. In July 2025, she received nab-paclitaxel 400 mg combined with fluorouracil 2 g via continuous infusion. During the fluorouracil infusion, she developed chest tightness and dyspnea, and fluorouracil was discontinued. The regimen was then modified to nab-paclitaxel plus capecitabine, with capecitabine 1.5 g orally twice daily.

At subsequent reassessment, CT showed gastric antral wall thickening and bilateral adnexal masses, broadly similar to the post-cycle 2 scan, consistent with stable disease. Tumor markers also showed a downward trend (**Table 1**). Because of persistent renal impairment (serum creatinine 144.4 μmol/L) and recurrent symptoms, further chemotherapy was deemed unsuitable. After renewed discussion of the risk–benefit profile and alternative options, the patient and her family agreed to switch to immunotherapy (e.g., sintilimab) with supportive care. She received two cycles of sintilimab 200 mg over the following weeks. Approximately 2 weeks after the second cycle, she developed recurrent symptoms concerning for malignant gastrointestinal obstruction. Although palliative radiotherapy and supportive care were evaluated and recommended, she ultimately died of rapid tumor progression, cachexia, and multiorgan failure.

## Discussion

This case illustrates a prototypical diagnostic dilemma of advanced gastric cancer presenting initially with bilateral adnexal masses highly suggestive of ovarian metastases (suspected Krukenberg tumor). According to the literature, patients with ovarian metastases from gastric cancer frequently exhibit peritoneal dissemination, ascites, and elevated CA125 levels—clinical features that closely resemble those of primary ovarian cancer—thereby substantially increasing the preoperative misdiagnosis rate (6), which was consistent with the initial diagnostic direction in our patient. Nonetheless, the subsequent clinical course underscored the distinctive nature of this case, in which the diagnostic “evidence chain” was simultaneously weakened by multiple factors. First, the initial EGD demonstrated only nonspecific inflammation, rather than the more typical endoscopic finding of a definite mass or ulcerative lesion. Second, 18F-FDG PET/CT likewise failed to reveal an abnormal hypermetabolic focus in the stomach. In addition, because the patient faced an imminent risk of acute obstruction and was in the early postoperative recovery period, the feasibility and safety of prompt repeat endoscopic sampling were limited. Such patient-related delays in diagnostic work-up are not uncommon in gastrointestinal malignancies; prior studies have reported diagnostic delay in approximately 23% of patients with gastric cancer (7). Collectively, these real-world constraints compelled the clinical team to establish a provisional working diagnosis and initiate management based on incomplete evidence. In this context, ovarian cancer–directed therapy was chosen as an interim pragmatic approach to stabilize disease and symptoms, while the diagnostic work-up for a gastrointestinal primary remained ongoing.

This case highlights the limitations of relying on a “single negative test.” Poorly differentiated gastric adenocarcinoma, particularly tumors with signet-ring cell components, often exhibits submucosal growth or diffuse infiltrative spread, with a relatively intact mucosal surface, which can reduce the diagnostic yield of an initial EGD biopsy (8). Therefore, in patients with persistent imaging evidence of gastric wall thickening, or whose metastatic lesions show histologic and/or immunophenotypic features suggestive of a gastrointestinal origin, a more aggressive sampling strategy should be considered. This may include multipoint and deeper biopsies and, when appropriate, endoscopic ultrasound-guided tissue acquisition. A recent systematic review and meta-analysis reported a pooled diagnostic yield of 82.6% for endoscopic ultrasound–guided fine-needle aspiration (EUS-FNA) in suspected scirrhous gastric cancer, supporting its use when standard endoscopic biopsies are negative or nondiagnostic (9).

Likewise, a negative PET/CT result should not be regarded as sufficient to exclude gastric cancer. Because signet-ring cell carcinoma and diffuse-type gastric cancer are often rich in mucin and have lower tumor cell density, the primary lesion may demonstrate low or absent FDG uptake (10). Prior reports suggest that the sensitivity of PET/CT in these histologic subtypes may decrease to approximately 60%–75% (11, 12). However, a balanced interpretation is warranted, as 18F-FDG PET/CT may still provide clinically useful complementary information in selected Krukenberg-type presentations; notably, Usmani et al. reported a patient with an ovarian mass in whom 18F-FDG PET/CT identified an occult primary gastric carcinoma (13). In addition, Ali et al. showed that 18F-FDG PET/CT detected an additional 8% of previously unrecognized metastases during routine staging of gastric cancer, supporting its incremental value for whole-body staging in selected patients (14). Therefore, PET/CT should be interpreted within a multimodality diagnostic framework rather than in isolation. In the present case, persistent antral wall thickening on contrast-enhanced CT, bowel-obstruction symptoms, and a gastrointestinal-favoring immunophenotype warranted continued pursuit of a gastric primary despite the negative PET/CT findings. Accordingly, in an “ovarian cancer-like” pattern of peritoneal dissemination, PET/CT is best viewed as an adjunct for whole-body staging and, in selected cases, for primary-site localization, but a negative scan cannot safely exclude gastric cancer.

From a pathobiological perspective, diffuse-type and poorly differentiated gastric cancers—particularly those with signet-ring cell components—are more prone to serosal invasion and cellular exfoliation. Once free tumor cells enter the peritoneal cavity, they can survive in peritoneal fluid/ascites as clusters and establish peritoneal metastasis through sequential steps of adhesion, invasion, and colonization (15). Concurrently, tumor-derived soluble factors and extracellular vesicles (including exosomes) can remodel the peritoneal mesothelial barrier and extracellular matrix, creating a pro-adhesive and pro-fibrotic “pre-metastatic niche” that further facilitates implantation and outgrowth of metastatic deposits (16, 17). Under the influence of physiologic peritoneal fluid circulation and gravity, tumor cells are more likely to accumulate in the pelvis and adnexal regions (18). In addition, the ovarian surface undergoes repeated cycles of “disruption–repair” during ovulation; the resulting localized inflammatory and tissue-repair milieu—together with relatively rich blood supply and altered vascular permeability—may provide a favorable “soil” for the adhesion, survival, and colonization of disseminated tumor cells. Consequently, clinical and imaging findings may paradoxically show a relatively occult primary lesion while peritoneal and/or ovarian metastases are more conspicuous (19). It should be noted that Krukenberg tumors may also arise via lymphatic or hematogenous dissemination; however, in the present case, given the phenotype of peritoneal carcinomatosis, peritoneal seeding more plausibly explains the observed metastatic distribution.

The key lesson from this case lies in the need for a dynamic, iterative diagnostic mindset. The clinical value of this case lies less in the mere occurrence of a false-negative PET/CT result, which has been reported previously, than in the management implication that negative PET/CT findings can create false reassurance when contrast-enhanced CT, metastatic pathology, and the clinical course continue to support a gastric primary. In such situations, discordant data should prompt diagnostic reassessment rather than therapeutic anchoring. A provisional working diagnosis should not be equated with a final diagnosis. When the clinical course and treatment response are inconsistent with expectations—such as rapid progression after ovarian cancer–directed chemotherapy, persistently rising tumor markers, and no improvement in gastric wall thickening—these findings should be regarded as strong warning signals that warrant a “diagnostic time-out” and prompt re-evaluation. In such circumstances, diagnostic priorities should be promptly recalibrated, and attention should return to the central task of confirming the primary site.

Importantly, this case should not be interpreted as supporting therapeutic decision-making based on PET/CT negativity alone; rather, it illustrates the risk of over-weighting a negative PET/CT result when other clinical, imaging, and pathologic findings remain discordant. From the standpoint of treatment and prognosis, this case also illustrates the cascading consequence of diagnostic delay leading to a narrowed therapeutic window. Once the diagnosis was revised, management should have aligned with standard pathways for advanced gastric cancer, incorporating biomarker-based stratification (e.g., HER2, PD-L1, and MSI/dMMR when available). However, recurrent obstruction, hydronephrosis, and renal insufficiency substantially limited the feasibility and tolerability of platinum-based or intensified regimens, and even subsequent immunotherapy maintenance was unlikely to reverse the trajectory toward end-stage systemic failure. This course underscores that, in such complex cases, early and sustained multidisciplinary involvement, together with proactive supportive care (symptom control, nutritional support, and organ-function optimization), should be front-loaded to enable clinical decisions that better balance risks and benefits in accordance with the patient’s condition. A key limitation is that ovarian involvement was inferred from imaging and intraoperative findings, without histologic confirmation from an ovarian specimen.

In summary, three clinical pearls emerge from this case. First, in women presenting with peritoneal carcinomatosis and adnexal masses, Krukenberg tumor/occult gastric cancer should remain high on the differential even when the initial EGD and 18F-FDG PET/CT are negative. Second, discordance between the immunophenotype (or clinical trajectory) and the working diagnosis should trigger an early diagnostic “time-out,” with repeat and deeper tissue sampling (including repeat EGD with multipoint biopsies and EUS-guided sampling when appropriate). Third, early multidisciplinary coordination and patient-centered communication are essential to expedite definitive testing and minimize treatment mismatch in real-world, time-pressured scenarios.

## Data Availability

The original contributions presented in the study are included in the article/supplementary material. Further inquiries can be directed to the corresponding authors.
